# The 170ms Response to Faces as Measured by MEG (M170) Is Consistently Altered in Congenital Prosopagnosia

**DOI:** 10.1371/journal.pone.0137624

**Published:** 2015-09-22

**Authors:** Andreas Lueschow, Joachim E. Weber, Claus-Christian Carbon, Iris Deffke, Tilmann Sander, Thomas Grüter, Martina Grüter, Lutz Trahms, Gabriel Curio

**Affiliations:** 1 Dept. of Neurology, Campus Benjamin Franklin, Charité - University Medicine Berlin, Berlin, Germany; 2 Department of General Psychology and Methodology, University of Bamberg, Bamberg, Germany; 3 Physikalisch-Technische Bundesanstalt, Berlin, Germany; University of Toyama, JAPAN

## Abstract

Modularity of face processing is still a controversial issue. Congenital prosopagnosia (cPA), a selective and lifelong impairment in familiar face recognition without evidence of an acquired cerebral lesion, offers a unique opportunity to support this fundamental hypothesis. However, in spite of the pronounced behavioural impairment, identification of a functionally relevant neural alteration in congenital prosopagnosia by electrophysiogical methods has not been achieved so far. Here we show that persons with congenital prosopagnosia can be distinguished as a group from unimpaired persons using magnetoencephalography. Early face-selective MEG-responses in the range of 140 to 200ms (the M170) showed prolonged latency and decreased amplitude whereas responses to another category (houses) were indistinguishable between subjects with congenital prosopagnosia and unimpaired controls. Latency and amplitude of face-selective EEG responses (the N170) which were simultaneously recorded were statistically indistinguishable between subjects with cPA and healthy controls which resolves heterogeneous and partly conflicting results from existing studies. The complementary analysis of categorical differences (evoked activity to faces minus evoked activity to houses) revealed that the early part of the 170ms response to faces is altered in subjects with cPA. This finding can be adequately explained in a common framework of holistic and part-based face processing. Whereas a significant brain-behaviour correlation of face recognition performance and the size of the M170 amplitude is found in controls a corresponding correlation is not seen in subjects with cPA. This indicates functional relevance of the alteration found for the 170ms response to faces in cPA and pinpoints the impairment of face processing to early perceptual stages.

## Introduction

We spend a considerable amount of our lives looking at and scrutinizing human faces. This is not surprising because at least in vision these stimuli are of outstanding importance for successful social communication. For major cognitive functions like face processing it is of prime importance to identify and explicitly describe those computational steps which are realized by specialized neuronal ensembles (modules). This will deliver deep insight into cerebral function and biologically inspire technical applications which are still far off the efficiency of real brains.

Currently it is still controversial whether face processing is at least partially modularized. Partial means that for example neural mechanisms of recognition / identification are modularized whereas others, e.g. the evaluation of facial expression, are not. Evidence from brain imaging with selective activations in occipitotemporal cortex, namely the fusiform gyrus [1] and various case reports of acquired prosopagnosia (aPA), a deficit of familiar face recognition, e.g. caused by ischemic infarction are taken as evidence for a face selective module [2, 3]. Opponents of this view claim that faces are exceptional due to massive overlearning because this leads to activations in the same locations for artificial objects [4]. Also, in many instances acquired prosopagnosia is accompanied by general deficits of object recognition [5].

We understand modularity in a Fodorian sense [6]. A thorough discussion of this complex term can be found in Pernet et al. [7]. Based on this discussion a cognitive module is instantiated by a set(s) of neurons that carry out a single specific task if and only if they are confronted with a well-defined piece of information (here: certain aspects of a face). The operation of the module cannot be carried out elsewhere in the brain (by any other set of neurons). Consequently its impairment cannot be directly compensated for and the corresponding function gets lost (here: impaired recognition of familiar faces). This does not necessarily imply overt behavioural confinement for the individual as the loss of a specific modular function can be compensated for by existing neural machinery; e.g., a person can be reliably recognized by his/her characteristic gait and stance.

More recently, by elegantly combining fMRI and single cell recordings in macaque monkeys Tsao, Freiwald, Tootell and Livingstone [8] could identify an area in which 97% of all neurons under study were highly face selective with a mean face selectivity index of 0.9 which is close to being specific. A few cells also gave a significant response to clocks and round fruits probably because these stimuli share the feature roundness with faces. This finding is indicative of modular processing, but first its behavioural consequence has to be shown and second a correspondence in humans has to be revealed.

Over the last years a form of prosopagnosia has attracted attention that presents itself without any indication of damage to the brain [9]. Because of lifelong impairment and familial clustering [10, 11] this condition has been termed “congenital prosopagnosia”. The core deficit in cPA is the impaired recognition of familiar faces which has been used as the defining neuropsychological criterion, e.g. [12]. Evidently analysis of this condition may significantly advance our understanding of face processing.

However, impaired face recognition performance in congenital prosopagnosia (cPA) is not sufficient to decide whether this condition is caused by dysfunction in core structures of face processing or e.g. by deviant social behaviour like in autism with malfunctioning outside of a face processing system [13]. The majority of studies using functional magnetic resonance imaging were unable so far to show consistent differences in activations between persons with cPA and controls in brain regions, implicated in face processing, especially in the so-called core face system [14–16]. It is well conceivable that the alteration of a transient face processing step evades the slow BOLD response (see [17] for the same argument). More recently though fMRI reveals functional as well as structural differences between subjects with cPA and controls: Furl et al. could significantly relate face selectivity of fusiform gyrus as measured by fMRI and face identification ability as measured behaviourally in subjects with cPA and controls [18]. Another study [19] using diffusion tensor imaging reports that reduction of white-matter tracts in ventral occipitotemporal cortex of persons with cPA—compared to controls—correlates with low performance in face recognition. This is complemented by two studies of the same group which use different fMRI paradigms to probe face processing and reveal a functional dissociation between the so-called “core” regions of face processing and “extended” regions outside [20, 21]. Surprisingly, also methods with high temporal resolution (EEG, MEG) have failed to provide conclusive results in cPA [16, 22–27]. Specifically the N170 (and its magnetoencephalographic analogon, the M170) which is considered as the earliest face-selective evoked response [28–31] lose their face selectivity only in a subset of persons with cPA. To account for the variability of results it has been argued [24, 25] that cPA comprises separate entities with impairment of different stages of face processing.

Given the current evidence we hypothesize that in face processing an early processing step (in the Bruce and Young Model [32] the structural encoder) tagged by N/M170 which codes the characteristic basic configuration that constitutes a face, i.e. the regions of eyes mouth and nose and their geometrical relation, is modularized and a necessary step in a chain of processes that lead to successful face recognition/ identification. We further hypothesize that this step is dysfunctional in congenital prosopagnosia. The circumscribed nature of the neural deficit in cPA implies that a corresponding behavioural impairment should be confined to aspects of face processing that directly rely on configural processing, e.g. recognition / identification.

We provided behavioural evidence for impaired configural processing in cPA for the same sample of subjects who participated in the current study [33, 34].

According to our hypothesis of early modularized dysfunctional face processing as evinced by electrophysiology and in order to show task independence of the predicted result we implemented two different continuous target detection tasks. In the first experiment subjects had to respond to the direction of motion of a face or a house target. In the second experiment subjects had to respond to an individual famous face (e.g. Bill Clinton) or house (e.g. Brandenburger Tor) target, which was memorized before the beginning of the experiment. We are aware of the fact that non-invasive imaging techniques cannot prove modularization of certain processing steps of cognitive function. But—as in the case of face processing—they can add evidence to corroborate the hypothesis of modularity.

## Materials and Methods

### 2.1. Participants

The control group consists of 16 persons (15 right handed; 7 women, mean age = 32, *SD = 9*, range 21 to 51 years). The target group consists of 13 persons (12 right handed; 10 women, mean age = 35, *SD = 12*, range 15 to 60 years). All persons had normal or corrected to normal vision. The educational level was different between both groups. In the control group there are 16 persons with final secondary school examination. In the target group there are 6 persons with final secondary school examination and 6 persons with general certificate of secondary education. Pearson’s chi-square-test (p<0.001**). One subject still attended high school. For study inclusion it was required that the medical history of all participants was without signs of psychiatric and neurological illness or drug abuse. This was ensured in an extensive interview by the first author of the study (A.L.) who is a senior neurologist and the third author (C.C.C.) who is a psychologist. None of the participants had to be excluded from further analysis due to medical reasons. All participants gave their written informed consent. One participant was minor (15 yrs.) at the time of the conduction of the study. Here, the mother gave the written informed consent on behalf of her daughter. All written informed consents were recorded on paper. This study (including the use of magnetoencephalography and electroencephalography) has been approved by the ethics committee of the Charité, Campus Benjamin Franklin.

### 2.2. Stimulus presentation and task

**Fig 1:**
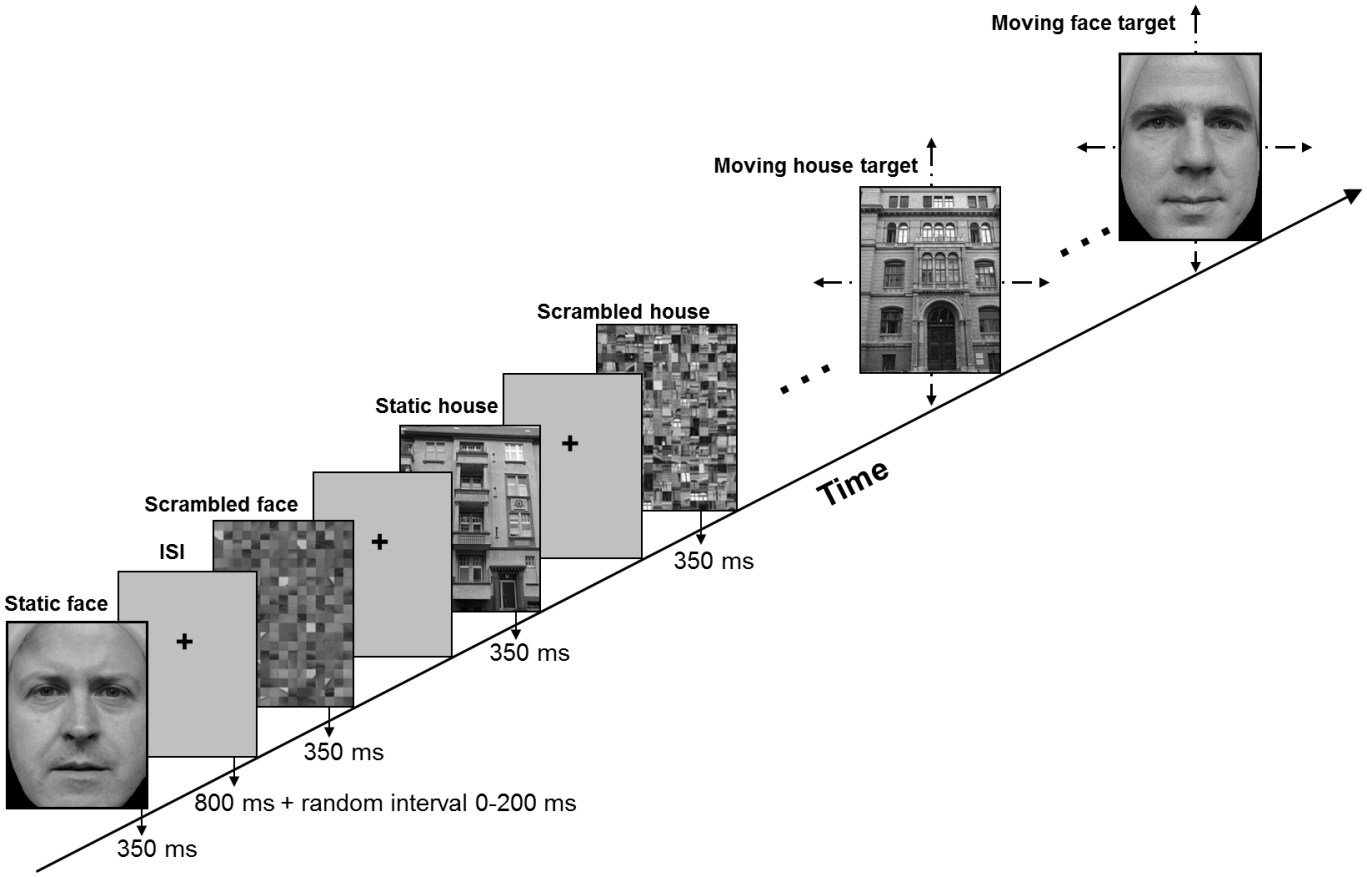
Stimuli and task for experiment 1 with two typical trials (face or house). The targets were inserted in the random sequence of faces and houses and persons responded differently to vertical or horizontal movement (illustrated by arrows).

In the experiment 1, participants viewed a random sequence of unknown face and house greyscale photographs (for details see Fig 1), 200 in each stimulus category. 85% (170/200) of the face stimuli and 85% (170/200) of the house stimuli were stationary non-targets. 15% (30/200) of faces and 15% (30/200) of houses were moving targets. Participants had to selectively respond to the target stimuli (for details see below). The entire experiment was divided into two blocks with a short break in between.

A non-target trial went on as follows: first, a face or a house stimulus appeared for 350ms, followed by an interstimulus interval of variable length between 800 and 1100ms due to a fixed interval of 800ms and a randomized interval between 0 and 200ms. The purpose of the randomization was to prevent synchronization of the alpha rhythm with the heartbeat. Second, an individually scrambled version of the face or the house was shown, again for 350ms. Scrambling was realized in the usual way by first dividing the face or house stimulus in squares that were randomly rearranged. The main purpose of the scrambled stimuli was to serve as difference stimuli in a localization procedure, but they are irrelevant in the context of the present analysis. The scrambled stimulus was also followed by a variable ISI which finished the trial.

A target trial was identical in timing and stimulus structure to a non-target trial, except for the fact that the face or the house stimulus could now move in vertical or horizontal direction which had to be indicated by key press with the left or the right index finger (motion discrimination). A motion sequence is detailed as follows: first, the stimulus was presented in the centre, second it randomly appeared at a position to the left/ right or up/ down of the initial position. Shift size was approximately 1 degree of visual angle. Third, the stimulus reappeared at the centre. Presentation time at all three positions was 75ms. Such a sequence creates the well-known illusion of apparent movement.

The rationale for this task was to have minimal impact on face and object processing in ventral occipitotemporal cortex because motion is analysed in area V5 being well removed from the fusiform face area [35].

**Fig 2:**
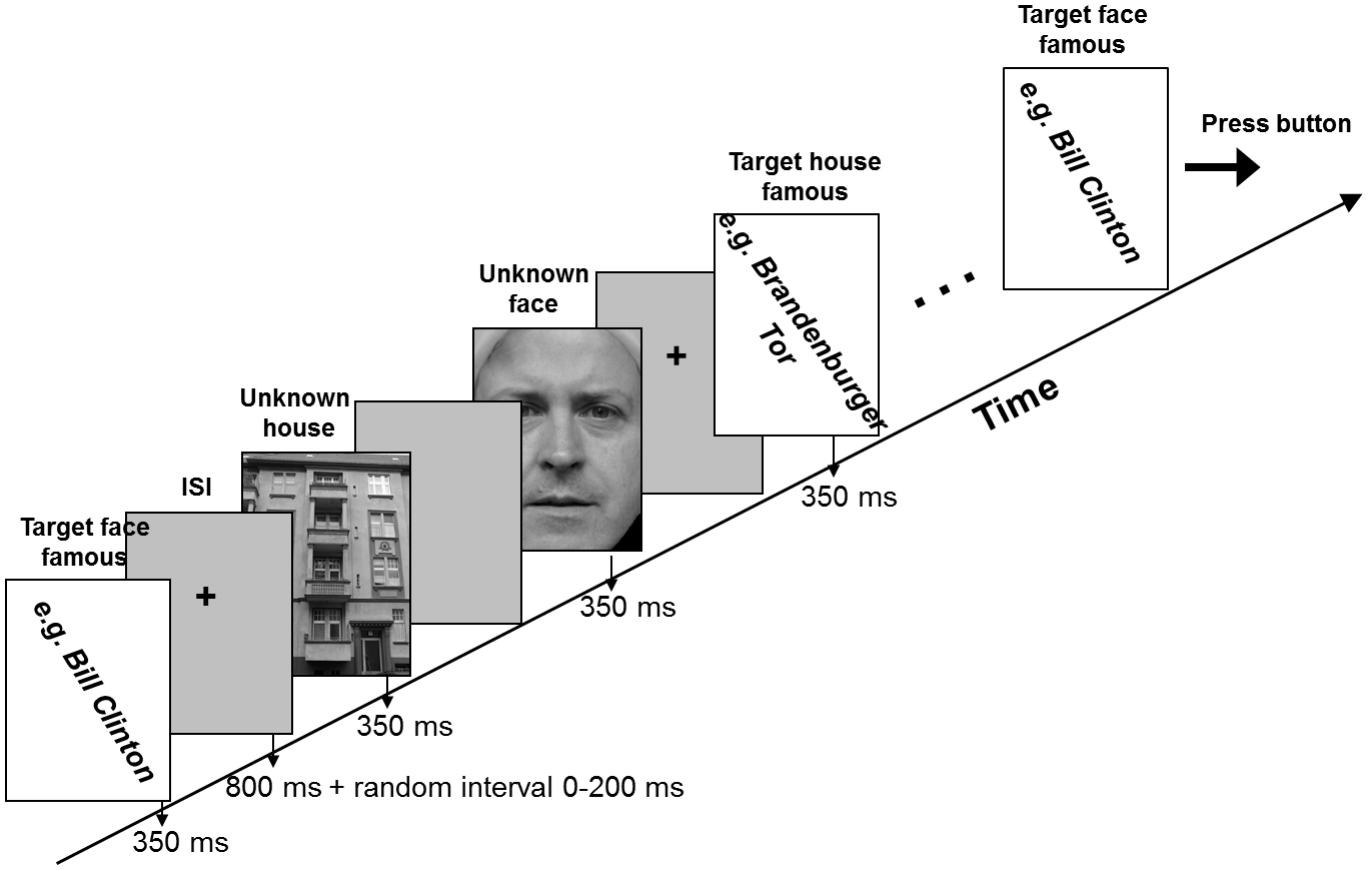
Stimuli and task for experiment 2. In contrast to experiment1 persons now had to respond to a single famous face or house which was inserted in the random sequence of unknown faces and houses. Because of copyright issues no examples of famous faces (e.g. Bill Clinton) and houses (e.g. Brandenburger Tor) are shown.

The main purpose of experiment 2 was to replicate the results of experiment 1 with a task of different cognitive load. It should be noted that experiment 1 is an attentive fixation task with no operation explicitly required to be performed on facial attributes since participants had to respond to the direction of motion. In experiment 2 subjects had to respond exclusively to a rare famous face or famous house target being inserted in a random sequence of frequent unfamiliar faces and houses (non-targets), for details see Fig 2. The reason to use unfamiliar non-targets was to exclude a priori any confounding due to the fact that the neural representation of familiar faces is hypothesized to be dysfunctional in congenital prosopagnosia. The timing of this experiment was identical to that of experiment 1 except for the fact that no scrambled stimulus was shown. In 16 subsequent blocks participants alternately had to respond to a face or to a house target. The face and the house target appeared 5 times each, amongst 25 unfamiliar faces and houses. Thus a single block consisted of 60 stimuli adding up to 16 x 60 = 960 stimuli over the entire session. In total, 400 unfamiliar faces and 400 unfamiliar houses were shown which gave 200 stimuli per condition (unfamiliar / familiar face or house) to average over. To maximize target recognisability–crucial for the persons with cPA—a set of 10 potential face and house targets was shown prior to the experiment. Each subject was asked to choose one famous face or house which he thought to recognize best. This face or house target was subsequently used. A further purpose of the second experiment was to replicate results from Lueschow et al. [36] and Furey et al. [17] according to which the M170 is not modulated by selective attention and we hypothesized that also in the present experiment this should be the case.

### 2.3. Data acquisition and artefact reduction

EEG and MEG were measured simultaneously in an electrically and magnetically shielded room. EEG was recorded from 27 electrodes attached to a cap (positions: O1, O2, PO9, PO10, T5, T6, T1, T2, T3, T4, Pz, P3, P4, CP5, CP6, Cz, C3, C4, Fz, Fc1, Fc2, Fc5, Fc6, F3, F4, F7,F8) using a reference electrode on the nose. The EEG-system had been developed by the Physikalisch-Technische Bundesanstalt Berlin. Electrodes were standard sintered silver/silver chloride electrodes, fixed on a cap (Easy Cap) at coordinates of the 10-20-system. EEG and EOG electrode impedances below 50kΩ were found to be sufficient. This was due to the high input impedance of 20mΩ of the EEG amplifier used and its low noise of 6.5nVHz^-1/2^ [37].

The magnetic fields were recorded with a 93-channel whole-head MEG system (Eagle Technology^TM^, ET160). Axial gradiometers with a baseline of 5cm were employed. Vertical and horizontal electrooculograms were recorded.

EEG and MEG were digitized with a sampling rate of 500Hz and a band-pass filter of 0.1–200Hz. Offline, the data were down-sampled to 250Hz and band-pass filtered with -3dB attenuation reached at 0.5 and 40Hz and a roll-off of 10dB per octave between 0.5 and 40Hz. An ICA was applied to EEG and MEG in an analogous fashion. This procedure has been proven to be especially effective in removing artefacts caused by the magnetic field of the heart in MEG data [38].

In addition to the ICA correction, EOG-artefact contaminated epochs were excluded using BESA (*Brain Electrical Source Analysis*, *MEGIS Software*,[39]). We identified eye-movement and blink artefacts manually for each subject by looking for deflections in the EOG channel. Depending on amplitude and duration triggers were set. The trials who met these criteria were eliminated. ERP’s and ERF’s were calculated over 650ms including a pre-stimulus baseline of 350ms.

### 2.4. Statistical analysis

In EEG the analysis was based on an averaged reference which was computed within BESA. Statistical comparisons between groups and stimuli were conducted for electrodes P7/P8 = T5/ T6 and PO9/PO10.

Rossion and Jacques (2008) in their review on the N170 detailed which electrodes are most sensitive to detect the typical face related deflection between 140 and 200 ms [40]. These are 4 pairs of lateral occipito-temporal electrodes (P7/P8 = T5/T6, PO8/PO7, P10/P9, PO10/PO9). As a matter of fact in all EEG studies on the topic of congenital prosopagnosia known to the authors one or more electrode pairs from this selection have been used, mostly P7/P8.

In MEG 18 physiologically motivated channels were chosen over left and right occipito-temporal cortex. To exclude any investigator driven bias an automatized routine (written in *Interactive Data Language*, *RSI*) searched within this subgroup for the largest M170 (this component was assumed to occur in an interval between 140 and 210ms) and its direct neighbours. Thus a spatial average was computed over 3 to 5 channels prior to computation of the individual trial based average.

There are two reasons why the automatized channel selection cannot be applied to EEG the main being the fact that the conventional 10–20 system is defined with respect to head coordinates whereas the location of the MEG channels is fixed with respect to the dewar which on the other hand precludes a fixed selection of channels. Second, the channel density is lower in the 10–20 EEG system which reduces the variability between sensors (electrodes). Because of the resulting larger spacing we did not consider it appropriate to compute a spatial mean for signals from different electrodes.

Peak latency and amplitude of face- and house evoked ERF/ ERP (N/M170) were evaluated by use of a mixed effect ANCOVA with factors stimulus and hemisphere (within subjects), group (across subjects) and age as covariate. Significance was assumed for *p*<0.05. In order to compare the time course and the magnitude of categorical (face minus house) differences in subjects with cPA and controls a running ANCOVA with factors stimulus and hemisphere (within subjects), group (across subjects) and age as covariate was performed for time intervals of 4ms according to down-sampling, the initial sampling rate being 500Hz. The hypothesis was that the interaction of stimulus and group should become significant in coincidence with the maximum of the categorical difference in controls minus the categorical difference in subjects with cPA (“double difference”).

In addition to the group statistics discriminant analysis (Fisher) using bilateral M170 latency and amplitude values as variables was used to classify individual subjects as belonging to the group of prosopagnosics or controls.

A generalized linear model (GLM) was used to compare results of the neuropsychological assessment between groups. Recognition performance was quantified by the proportion of correct answers (percent correct). Our self-made test (Famous Face/House Recognition Test; for a detailed description see S1 Text) allows to compute the d-prime which is a common way to eliminate response bias. The d-prime is the z- transform of hits corrected by false alarms (here: the proportion of unknown faces or houses that were erroneously classified as famous). To evaluate the brain-behaviour-relationship Pearson correlations were computed between M170 amplitude and performance (percent correct, d’).

## Results

We explored face processing in 16 controls and 13 persons with cPA. Subjects who entered the present study with strongly suspected diagnosis of cPA had taken part in a semi-structured interview as has been described in [41]. The interview was not strictly formalized except for the fact that a defined set of key questions had to be asked which was loosely embedded into the course of the interview. If these questions were answered positively than a subject was given the diagnosis of congenital prosopagnosia. The most discriminative symptoms for the diagnosis of prosopagnosia are listed in Table 1 in [41] and as follows: the item which proved to be a diagnostic hallmark of prosopagnosia is the uncertainty about the familiarity of a face which leads to false-negative and false-positive face recognition events (i.e. even close family members are overlooked when met in an uncommon situation or strangers are confused with familiar persons). Also subjects with cPA require a substantially prolonged face recognition and face learning time (the time being significantly longer than socially accepted; which for example irritates colleagues at the workplace). Subjects cannot remember the onset of symptoms, they regard gaze contacts as not necessary for social contact and they use adaptive behavior (e.g. avoid critical situations which could lead to difficulties to recognize familiar persons). The development of adaptive behavior points to a long-standing perceptual deficit. Also explicit compensatory strategies are used to recognize familiar persons (e.g. hair style and hair color).

### 3.1. Neuropsychological assessment reveals face-selective impairment in prosopagnosics

In a large variety of neuropsychological tests assessing major cognitive functions both groups were indistinguishable (for details see S1 Text, S1 and S2 Tables). In three standardized tests of face processing, the Benton Facial Recognition Test [42], the Warrington Recognition Memory Test [43] and the recently developed Cambridge Memory Test for Faces [12] persons with cPA scored significantly lower than normal controls thus meeting the definition of prosopagnosia (Table 1). In the CMTF recognition of subjects with cPA was also impaired for inverted faces. For a detailed description of different tests of face processing cf. S1 Text.

**Table 1:** Face recognition tests for controls and individuals with Prosopagnosia.

Test	Controls (mean[iqr])	cPA (mean[iqr])	sign.[Wald-Chi-square]
BFRT (%)	88.66[10.18]	80.91[10.19]	**<.001[15.144]**
RMT-F (%)	90.88[5.50]	84.92[12.00]	**.004 [8.525]**
CMTF upright faces (%)	74.81[16.66]	56.73[15.28]	**<.001[18.765]**
CMTF inverted faces (%)	59.17[12.50]	50.69[10.43]	**.009[6.914]**

**Fig 3:**
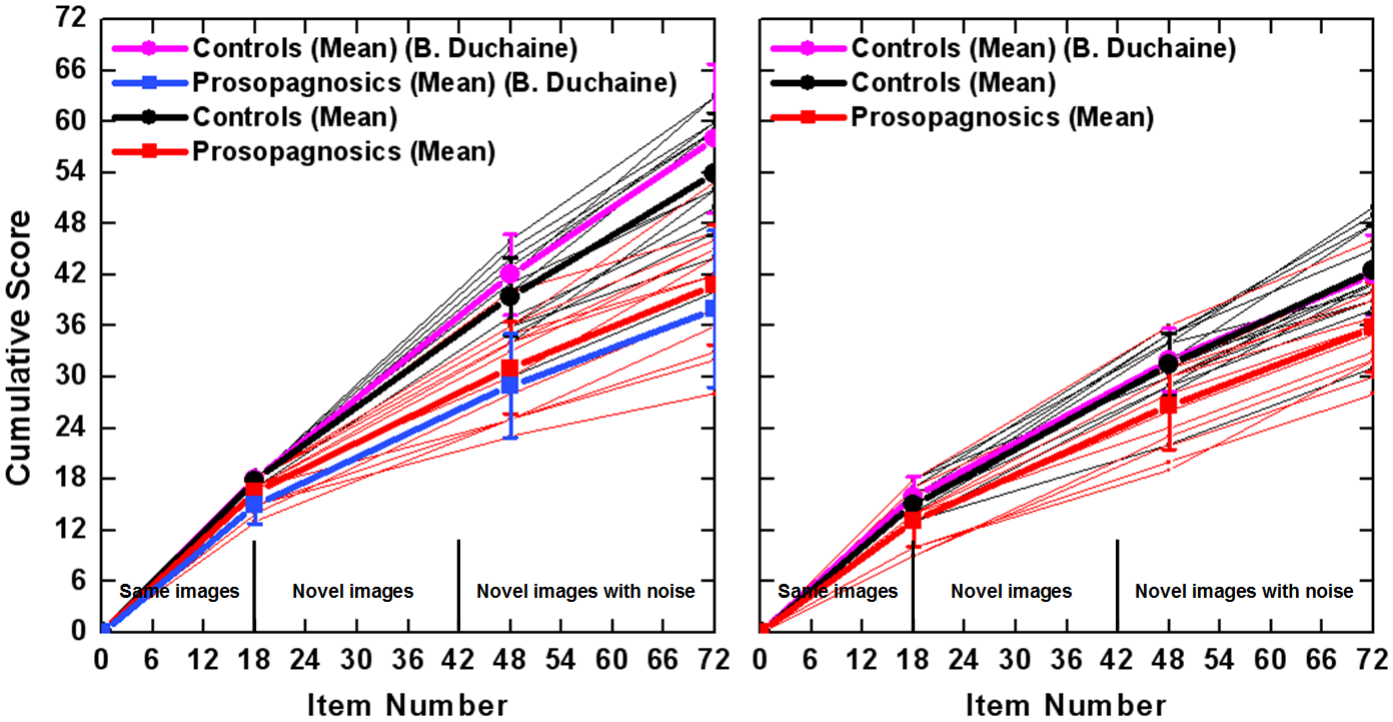
Comparison of the results of the Cambridge Memory Test for Faces (CMTF) in this study to the original results to standardize the CMTF [12]. Summary scores from Appendix C from [12] were used with permission by Brad Duchaine. The present Figure was prepared in the style of Figs 4 and 5 from [12]. For the so called cumulative score the number of faces that were correctly recognized in the three sections of the CMTF were added up from the first to the third part. In the first part (called same images) the maximal number of recognized faces is 18, for the second part 30 (i.e. the cumulative score of part 1 and 2 is 48) and for the third part 24 (for more details see Duchaine and Nakayama 2006). Good to perfect correspondence between the present data and those from [12] can be seen for upright (left panel) and inverted faces (right panel). Note, that in the original study people with cPA did not perform the inverted version of the test.

Fig 3 illustrates that recognition performance of persons studied here (16 controls and 13 subjects with cPA) is in excellent accordance with the groups examined to standardize the CMTF (50 controls, 8 persons with cPA) [12].

**Fig 4:**
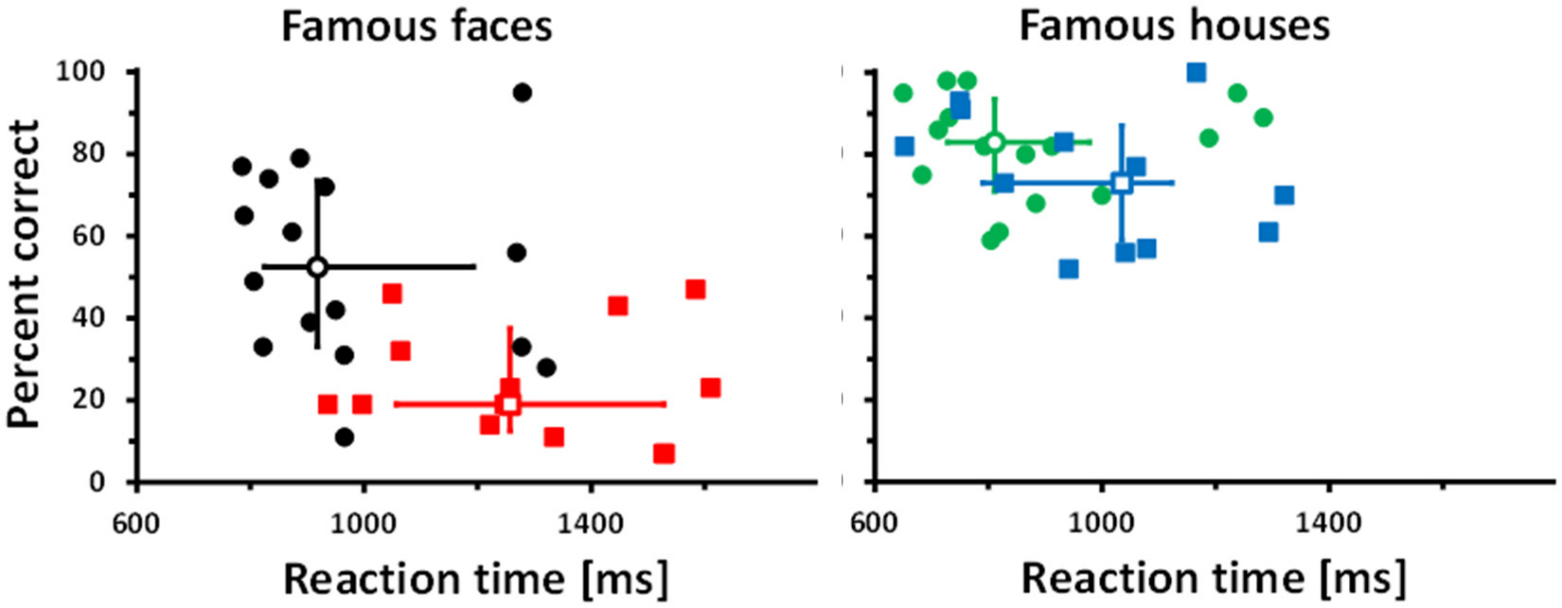
Famous face/house recognition test. Persons with cPA show significantly reduced accuracy and significantly extended reaction times for face recognition (red squares) compared to controls (black circles). For object recognition behaviour is indistinguishable between groups (cPA: blue; controls: green). Group medians are represented by open symbols. Error bars indicate the IQR.

Finally in a self-made test (FFHRT) persons had to indicate whether a given stimulus was famous or unknown in a random sequence of faces and houses. Face recognition was impaired in persons with cPA but object (house) recognition was indistinguishable between both groups, for details see Fig 4.

Generalized linear model with factors group and stimulus revealed a significant interaction of group x stimulus (dependent variable accuracy): p = 0.042 (*Wald-Chi-square* = 4.631). Direct comparisons showed a significant difference in performance of the two groups for faces (p = 0.008 (*Wald-Chi-square* = 7.041)) but indistinguishable performance for objects (*p = 0*.*615 (Wald-Chi-square = 0*.*253)*; for details see S2 Table). A comparison of reaction times (RTs) yields similar results: RTs of persons with cPA are significantly slower for faces (*p = 0*.*040 (Wald-Chi-square = 4*.*199*), but indistinguishable for houses (*p = 0*.*309 (Wald-Chi-square = 1*.*035)*).

Results for d-prime instead of percent correct are similar (for details see S1 Fig, upper half). Accuracy measures for famous face and object recognition are not correlated in both groups (for details see S1 Fig, lower half).

**Fig 5:**
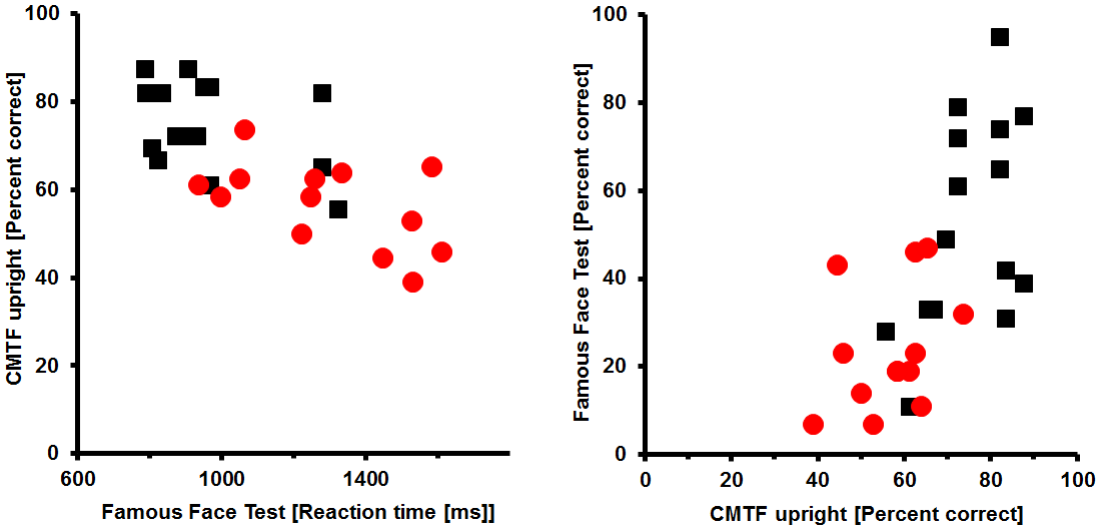
Scatter plot relating performance in two tests of face processing (CMTF versus FFHRT). Performance of persons with cPA is illustrated by filled red circles; performance by unimpaired controls is illustrated by filled black squares.

Performance in the different tests of face processing was related against each other using Pearson correlations. The most reliable correlations were seen for the CMTF and a self-made test of face processing (Fig 5), the correlation coefficient being 0.707 (p < 0.001; left half of Fig 5) and 0.662 (p < 0.001; right part of Fig 5). The remaining correlation measures are displayed in S3 Table).

### 3.2. Latency and amplitude of M170 distinguish prosopagnosics from unimpaired persons

**Fig 6:**
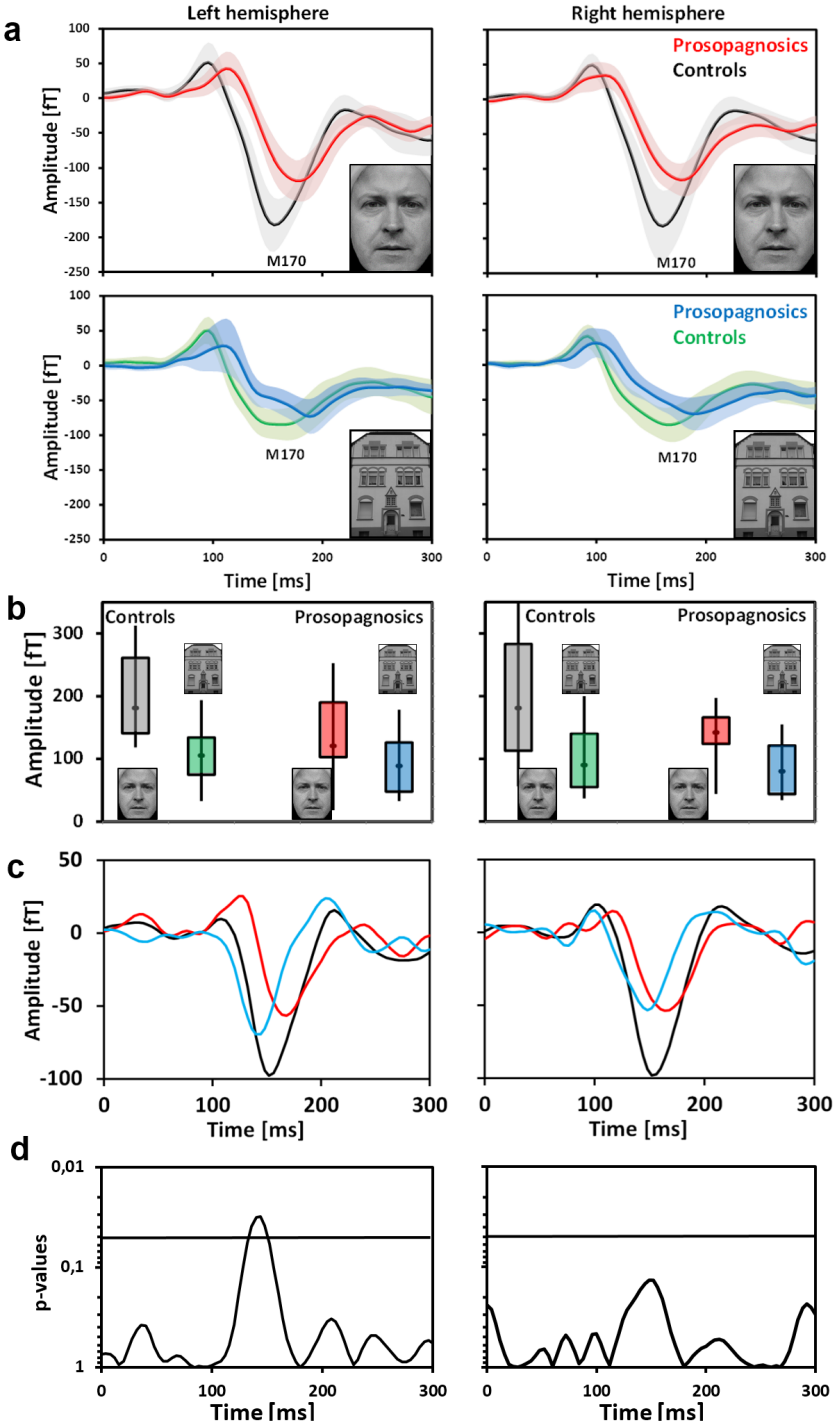
MEG result. Grand averaged curves (A) reveal significant differences of amplitude and latency (left) between persons with cPA and controls for faces and indistinguishable evoked responses for objects. The semitransparent confidence surfaces illustrate 1 SD. (B) Categorical differences (faces vs. houses) of M 170 peak amplitudes are represented by box plots representing the median, the lower and the upper quartile as well as the minimum and maximum values. (D) Categorical difference curves: faces minus houses (red: persons with cPA, black: controls, blue: categorical difference cPA minus categorical difference controls “double difference”). (E) Temporal evolution of interaction term (stimulus with group). The peak coincides with the peak of the “double–difference curve” (bilaterally).

In the neurophysiological experiment MEG and EEG were recorded simultaneously (see Methods). Fig 6A (upper half) illustrates the main result: the mean amplitude of the M170—evoked by faces—is reduced by 29.6% over right occipitotemporal cortex in the target compared to the control group (-138fT vs. -196fT) and by 30.3% over left occipitotemporal cortex (-138fT vs. -191fT). Mean peak latency of M170 is delayed by 8ms over the right hemisphere in the target group (167ms compared to 175ms) and by 16ms over the left hemisphere (161ms compared to 177ms). The data were subjected to an ANCOVA with within-subject factors hemisphere and stimulus, between-subject factor group and covariate age which showed a significant interaction between stimulus and group for amplitudes (p = 0.005, F = 9.636), but not for latencies (p = 0.947, F = 0.005). No other interaction was significant or nearly significant. The physiological interpretation of this interaction is that it is caused by the reduced M170 amplitudes to faces in subjects with cPA. This can be directly taken from Table 2 which displays the results from the ANCOVA in detail: main effects for factor group are significant bilaterally for the amplitude of the M170 to faces but not to houses. Main effects for factor stimulus (face selectivity) are highly significant for controls and significant for subjects with cPA only on the left hemisphere.

**Table 2:** Detailed results from the mixed ANCOVA are shown for experiment 1; MEG: upper half; EEG: lower half.

		Factor stimulus			Factor group		Stimulus X Group	
		(face vs house)			control vs cPA			
		p	F		p	F	p	F
**M170 amplitude (left)**	**controls**	**0.41x10** ^**-4**^	**23.065**	**faces**	**0.001**	**13.048**	**0.039**	**4.385**
	**cPA**	**0.034**	**5.066**	**houses**	**0.174**	**1.958**		
**M170 amplitude (right)**	**controls**	**0.001**	**12.953**	**faces**	**0.018**	**6.362**		
	**cPA**	**0.297**	**1.1358**	**houses**	**0.393**	**0.755**		
**M170 latency (left)**	**controls**	**0.278**	**1.220**	**faces**	**0.010**	**7.751**	**0.976**	**0.001**
	**cPA**	**0.899**	**0.017**	**houses**	**0.549**	**0.369**		
**M170 latency (right)**	**controls**	**0.880**	**0.023**	**faces**	**0.284**	**1.195**		
	**cPA**	**0.003**	**11.386**	**houses**	**0.059**	**3.903**		
**N170 amplitude (PO9)**	**controls**	**0.71x10** ^**-4**^	**21.189**	**faces**	**0.376**	**0.812**	**0.600**	**0.276**
	**cPA**	**0.021**	**6.145**	**houses**	**0.369**	**0.835**		
**N170 amplitude (PO10)**	**controls**	**0.95x10** ^**-4**^	**20.272**	**faces**	**0.833**	**0.045**		
	**cPA**	**0.019**	**6.322**	**houses**	**0.797**	**0.068**		
**N170 latency (PO9)**	**controls**	**0.636**	**0.229**	**faces**	**0.262**	**1.314**	**0.171**	**1.900**
	**cPA**	**0.180**	**1.909**	**houses**	**0.423**	**0.663**		
**N170 latency (PO10)**	**controls**	**0.135**	**2.358**	**faces**	**0.968**	**0.002**		
	**cPA**	**0.722**	**0.130**	**houses**	**0.451**	**0.586**		

For latency the non-significant interaction between stimulus and group is obviously due to the fact that a significant main effect of factor group was only obtained over the left hemisphere for latency of the face evoked M170.

The results of the first experiment were replicated by the second experiment. This is complemented by S2 and S3 Figs as well as S4 Table which reveal that amplitude and latency measures are highly correlated between the two experiments. As this experiment does not show any effect of attention on the 170ms response to faces and houses (i.e. responses with and without attention were statistically indistinguishable) which corroborates earlier results from our group as well as others [17, 36] we decided to only present results when attention was focussed on the stimulus categories (see Table 3 for details). Now the interaction between factors stimulus and group is significant for amplitude as well as for latency. Main effects for factor stimulus are highly significant for controls as well as significant for subjects with cPA which reveals that face selectivity is preserved in subjects with cPA although substantially reduced by approximately 45% which can be taken from the box plots of Fig 6B.

**Table 3:** Detailed results from the mixed ANCOVA are shown for experiment 2 (stimuli attended); MEG: upper half; EEG: lower half.

		Factor stimulus			Factor group		Stimulus X Group	
		(face vs house)			control vs cPA			
		p	F		p	F	p	F
**M170 amplitude (left)**	**controls**	**0.16x10** ^**-5**^	**29.064**	**faces**	**0.004**	**9.713**	**0.022**	**5.410**
	**cPA**	**0.013**	**7.106**	**houses**	**0.053**	**4.077**		
**M170 amplitude (right)**	**controls**	**0.1x10** ^**-6**^	**32.221**	**faces**	**0.004**	**9.851**		
	**cPA**	**0.008**	**7.667**	**houses**	**0.440**	**0.612**		
**M170 latency (left)**	**controls**	**0.144**	**0.872**	**faces**	**0.008**	**8.20**	**0.033**	**4.691**
	**cPA**	**0.806**	**0.061**	**houses**	**0.737**	**0.115**		
**M170 latency (right)**	**controls**	**0.137**	**0.188**	**faces**	**0.01**	**7.678**		
	**cPA**	**0.843**	**0.039**	**houses**	**0.689**	**1.472**		
**N170 amplitude (PO9)**	**controls**	**3.38x10** ^**-4**^	**16.503**	**faces**	**0.884**	**0.022**	**0.401**	**0.710**
	**cPA**	**0.200**	**1.744**	**houses**	**0.796**	**0.068**		
**N170 amplitude (PO10)**	**controls**	**0.003**	**10.682**	**faces**	**0.845**	**0.039**		
	**cPA**	**0.047**	**4.387**	**houses**	**0.570**	**0.330**		
**N170 latency (PO9)**	**controls**	**0.098**	**2.922**	**faces**	**0.403**	**0.724**	**0.732**	**0.118**
	**cPA**	**0.864**	**0.030**	**houses**	**0.768**	**0.089**		
**N170 latency (PO10)**	**controls**	**0.315**	**1.047**	**faces**	**0.952**	**0.004**		
	**cPA**	**0.748**	**0.106**	**houses**	**0.274**	**1.251**		

**Fig 7:**
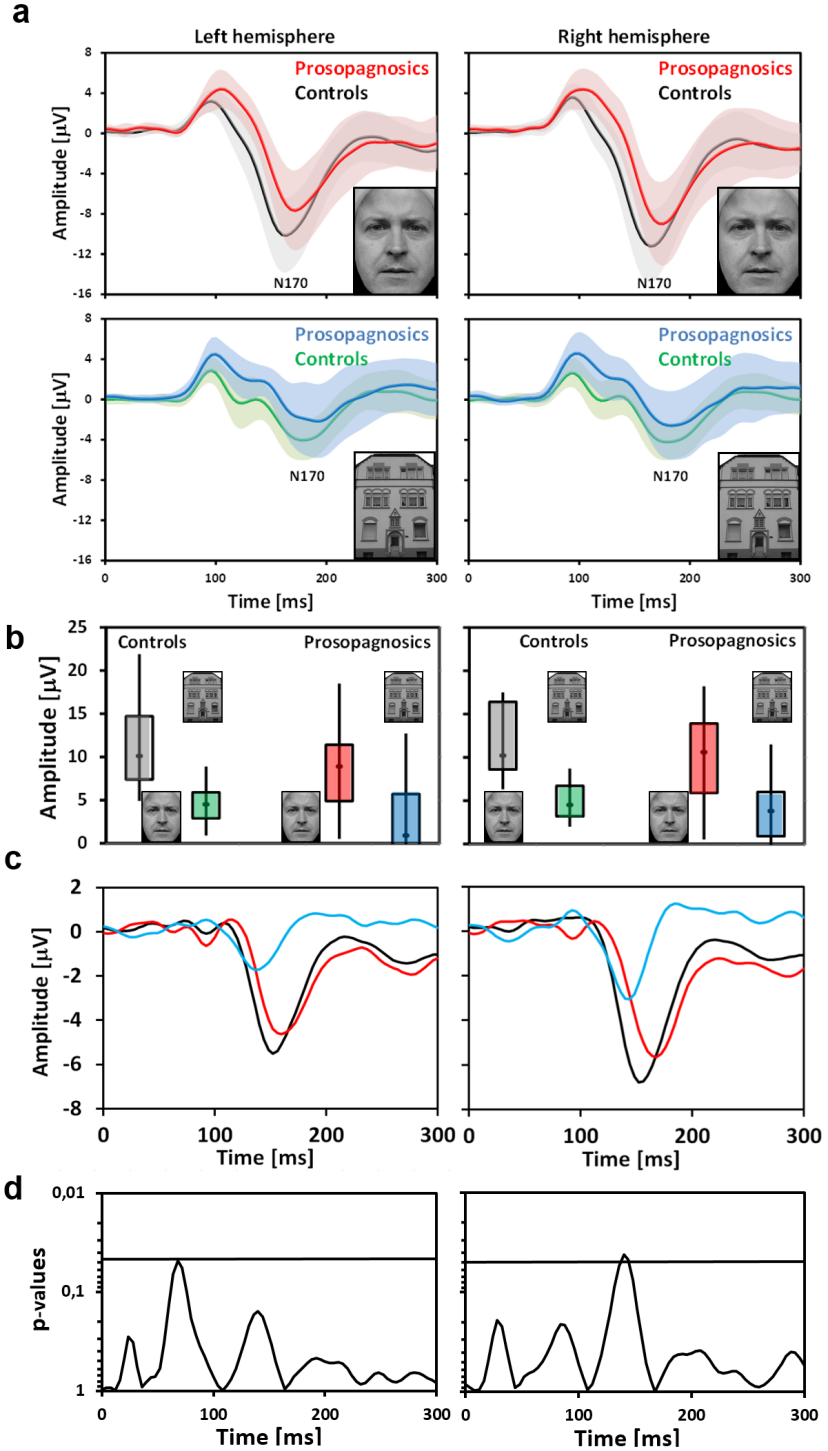
EEG result. In contrast to MEG amplitudes and latencies of grand averaged curves (A) are statistically indistinguishable both for faces and for houses. (B) Representation of categorical differences (faces vs. houses) by box plots. (C) Categorical difference curves: faces minus houses (red: persons with cPA, black: controls, blue: categorical difference cPA minus categorical difference controls “double difference”). (D) Temporal evolution of interaction term (stimulus with group). The peak coincides with the peak of the “double–difference curve” (right hemisphere).

Most interestingly, in contrast to MEG EEG (analysed with a mixed effect ANCOVA analogous to the MEG data) does not detect significant differences between both groups (lower halves of Tables 2 and 3 and Fig 7). The interaction between stimulus and group for electrodes PO9 and PO10 which are assumed to be most sensitive for the N170 was neither significant for amplitudes nor for latency in both experiments. This coincides with insignificant main effects for factor group. Except for electrode PO9 in the first experiment preserved face selectivity is revealed by EEG. The same result was obtained at electrodes T5 and T6. The difference between EEG and MEG can be interpreted such that these methods differentially capture sources in occipitotemporal cortex.

In order to study the temporal evolution of categorical (face minus house) differences and to compare them between the two groups a running mixed ANCOVA with within-subject factors hemisphere and stimulus, between-subject factor group and covariate age (see Statistical analysis) was applied to the MEG and EEG data (the categorical difference curves for the two groups are displayed in Figs 6C and 7C (red versus black); the blue curves result from subtracting the difference curve for subjects with cPA from that of controls: “double-difference”). In this analysis the interaction term (stimulus with group) both for EEG and MEG becomes significant in precise coincidence with the peak of the “double-difference” curve (MEG: p = 0.011; F = 6.676; EEG: p = 0,015; F = 6.156). This peak occurs almost 20ms prior to the M170 in controls (latency of 150 compared to 167ms (left) and 142 compared to 161 (right)). The same is true for EEG.

If the running ANCOVA is applied separately for both hemispheres (Figs 6D and 7D) the interaction term (stimulus with group) becomes significant for MEG on the left side and approaches significance on the right side, the reason for the latter probably being the fact of pronounced variance due to artefacts and not physiological causes as the grand averages are almost identical on both sides (see Fig 6C). For EEG the data seem less clear: on the right side the interaction becomes significant in coincidence with the peak of the “double difference”; on the left there is no significant effect in the same time range (it can be taken from the difference curves that the double difference grand average is small in absolute terms, Fig 7C, left inset).

A further statistical analysis, discriminant analysis (Fisher) using bilateral M170 latency and amplitude values as variables correctly classified 89.7% of all persons (11 out of 13 persons with cPA and 15 out of 16 controls).

**Fig 8:**
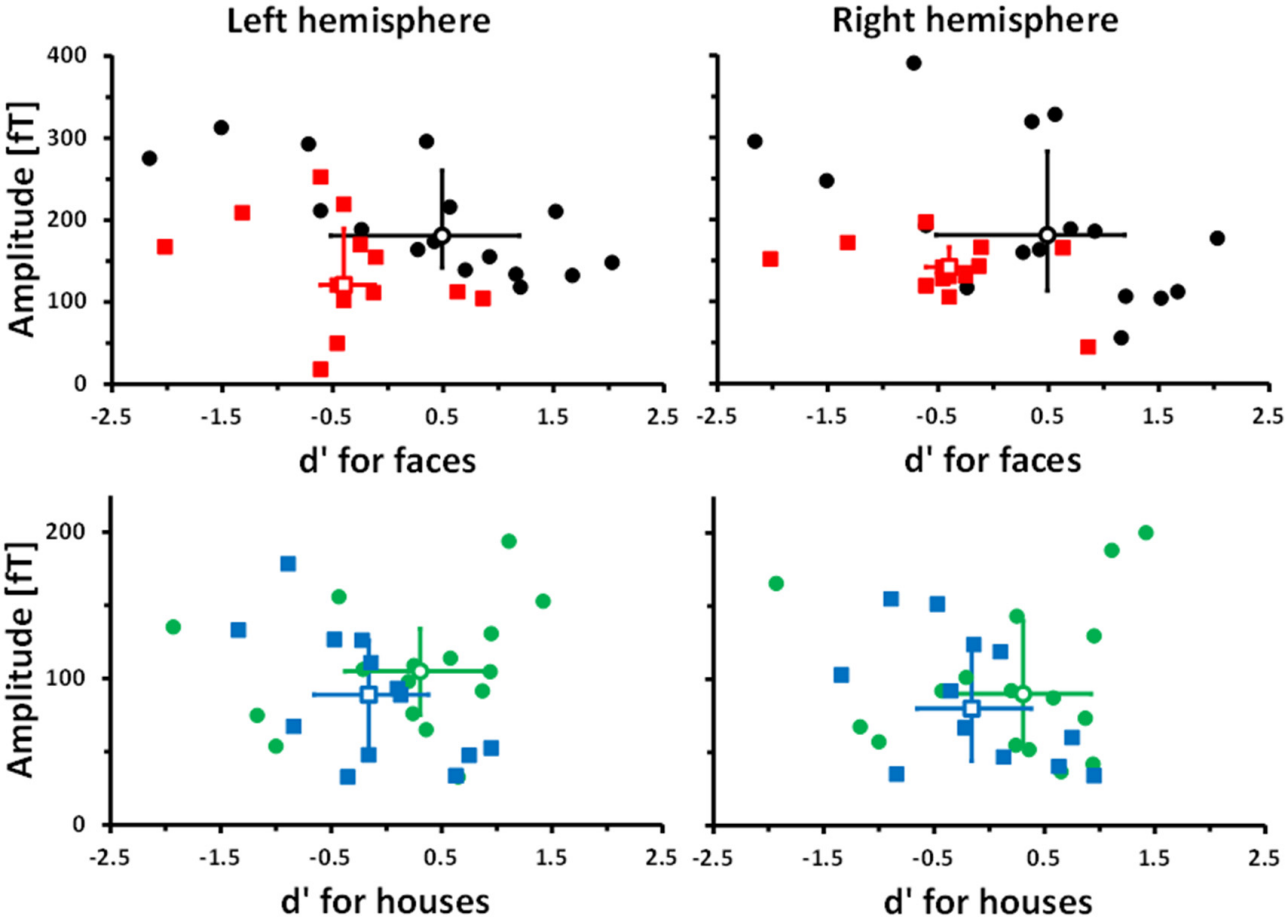
Scatter plots: M170 amplitude versus dprime (d’). For colour coding cf. Fig 2. Table 2 displays the statistics. Open symbols represent medians; error bars show IQR’s.

Finally a brain-behaviour correlational analysis revealed the following: face recognition performance measured as percent correct was not significantly correlated with face evoked M170 amplitude neither in controls nor in prosopagnosics. In order to eliminate a possible response bias the d-prime was computed (see Methods). This revealed a significantly negative correlation between d’ and the amplitude of the M170 evoked by faces in controls but not in subjects with cPA. For object recognition both performance measures (percent correct and d’) gave a significantly negative correlation with the amplitude of the house evoked M170 in subjects with cPA whereas there was no significant correlation in controls (for details see Table 4 and Fig 8).

**Table 4:** Brain-behaviour correlations. Bold numbers denote correlation coefficients and level of significance (numbers in brackets) for M170 amplitude vs. d’ (the z-transform of hits corrected by false alarms). The corresponding analysis with percent correct is presented by italicized numbers.

Amp M170 vs. d’	Correlation coefficient (significance)	
	Controls	Persons with cPA
Faces (left)	**-0.742 (<0.001)**	**-0.294 (0.329)**
	*-0*.*339 (0*.*199)*	*-0*.*257 (0*.*397)*
Faces (right)	**-0.561 (0.024)**	**-0.436 (0.136)**
	*-0*.*349 (0*.*185)*	*-0*.*265 (0*.*382)*
Houses (left)	**0.208 (0.440)**	**-0.622 (0.023)**
	*0*.*182 (0*.*499)*	*-0*.*607 (0*.*028)*
Houses (right)	**0.117 (0.666)**	**-0.512 (0.074)**
	*0*.*186 (0*.*489)*	*-0*.*809(<0*.*001)*

## Discussion

In this MEG/EEG study a group of 13 individuals with congenital prosopagnosia (cPA) who met objective neuropsychological criteria was compared with a group of 16 unimpaired controls in two subsequent experiments using different target detection tasks.

Both experiments (with slight deviations which are discussed below) showed that early face-selective responses (the M170) are significantly reduced in amplitude and significantly prolonged in latency in subjects with cPA. A comparison of the time course of categorical differences between both groups indicates a difference at 150ms, which is even prior to the peak of the M170. This is the first group level evidence for such an early alteration in cPA. Discriminant analysis (Fisher) based on bilateral amplitude and latency values of the face-evoked M170 revealed an almost complete separation (90% of all cases) of persons with cPA and controls. The reduction in amplitude and prolonged latency of the M170 are face-selective because object- (house) related responses are statistically similar between groups. The face selectivity of the impairment in congenital prosopagnosia is supported by extensive behavioural testing which did not reveal any impairment in object-related processing.

The detection of an early perceptually-based alteration in the sequence of transformations that lead to final recognition or identification of an individual face is important because it has been hypothesized that prosopagnosia results from problems at later stages of face processing [25, 44] In the framework of Bruce and Young 1986 these are the so-called face-recognition units (FRUs) which are characterized by the establishment of stable and view-point invariant representations of faces [32]. The present results together with behavioural results from two earlier studies with the same sample of subjects [33, 34] point to an interpretation that a perceptually-based (configural) analysis of critical facial information is a necessary prerequisite in the sequence of events that lead to successful recognition.

### 1. Why did experiment 1 and experiment 2 produce slightly different results?

The study results were evaluated statistically by a mixed ANCOVA which showed a significant interaction between stimulus (face versus house) and group (cPA versus controls) for amplitude in the first experiment and both for amplitude and latency in the second experiment (see Tables 2 and 3 for details). The physiological interpretation of this result is that it is caused by a significant decrease (approximately 30%) of the M170 amplitude to faces both in experiment 1 and 2 as well as to a significant delay (approximately 15ms) of the M170 peak bilaterally in experiment 2 in the cPA group. In experiment 1 the M170 peak was only clearly (approximately 15 ms) delayed over the left occipitotemporal cortex which caused the interaction to be insignificant for latency. Against the background of experiment 2 this is most likely due to the fact that the automatized determination of peak latency is error-prone especially in subjects with cPA because of dampened evoked activity with decreased amplitudes.

### 2. Which physiological explanation can account for the present results?

The delay in latency and the decrease in amplitude of the M170 in prosopagnosics can be explained in terms of the two component model of the 170ms response to faces developed by Bentin and colleagues [30, 45]. The model is supported by intracranial recordings of the N200 [46, 47]. It assumes a “holistic” process located more medially in the fusiform gyrus and a “part-based” process in more lateral parts of the occipitotemporal cortex. The term “holistic” refers here to the formation of the individual face Gestalt. This process is assumed to be based upon the evaluation of the relation of prominent facial features (parts) like eyes, mouth and nose—usually referred to as configural processing, an essential component of holistic processing—and their linkage into some higher-order facial representation, e.g. Maurer et al. 2002 [48]. The explicit nature of this representation is one of the enduring riddles in cognitive neuroscience. The holistic process is triggered by default (“primacy of holistic processing”). The part-based processes are only activated if face processing is impeded e.g. by presenting the face upside down (the “face-inversion effect”). The delay of the N170 to inverted faces is usually attributed to the hypothesis that part-based processing is more time consuming and therefore slower compared to holistic processing, e.g. Rossion et al. 1999, Sagiv and Bentin 2001, [45, 49].

One factor that could contribute to the partly discrepant result between MEG and EEG is their differential sensitivity to preferred radially oriented sources in the lateral temporal cortex and to preferred tangentially oriented sources in the fusiform gyrus. But, as will be discussed below, other factors could also have a significant contribution, e.g. sampling density due to different numbers of recording channels.

In the context of the two-component model the decrease in amplitude and the delay of the M170 can be directly explained by insufficient or absent activation of the presumably dysfunctional holistic processor in the fusiform gyrus in persons with prosopagnosia. It has to be stressed here that in this experiment face stimuli are viewed passively without any task required to be performed on them. Thus it can be expected that the 170ms response is dominated by mandatory holistic processing. Our hypothesis that faster holistic processing is insufficiently triggered in subjects with cPA has a direct correspondence with the result of the time course analysis of the face minus house (categorical) differences in persons with cPA compared to controls (the so-called “double difference” curves in Figs 6C, 6D, 7C and 7D. The peak of this curve precedes the peak of the 170ms response to faces by about 20ms in unimpaired subjects in EEG and MEG. Although the M170 is also significantly altered in subjects with cPA, in this group the M170 amplitude to faces is significantly higher compared to houses (preserved face-selectivity). This can be explained by a normal activation of a “part-based” process, preferentially tuned to faces, in lateral parts of the occipitotemporal cortex.

### 3. Is it possible to relate the 170 ms response to faces to behaviour?

In addition to the finding that early electrophysiological markers (especially the M170) are different between subjects with cPA and controls on a group level, differential brain-behaviour correlations between groups (cf. Table 2) indicate a functional relevance of altered early face processing. As face recognition is significantly degraded in adult subjects with cPA the underlying dysfunctional mechanisms cannot be adequately compensated for. One could therefore speculate that the result of the correlational analysis is indicative of the intrinsically modular organization of an early face processing stage.

It is interesting that the amplitude of M170 and face recognition are inversely related. Assuming that the amplitude of the M170 is directly related to the size of the underlying neural ensemble, one could consider this as a sign of sparse coding with smaller neuronal ensembles indicating higher coding efficiency. This result is paralleled by single unit recordings in monkeys [50] which show that sharpening of visual shape selectivity after training was accompanied by a reduced population response between 80 and 180ms. The demonstrated inverse brain-behaviour relationship reveals that the size of the amplitude of N and M170 is a complex measure being dependent not only on the tuning to a single class of visual objects (as is suggested by the conventional definition of face selectivity) but possibly also to the genetically determined individual “neural wiring”. To our knowledge so far no significant brain-behaviour relation has been established for N or M170 amplitude; see for example [51] for a recent EEG study with a negative result.

One factor that might have masked such a relation is a strong response bias.

When we first analyzed a possible brain-behaviour relation by the use of percent correct as a performance measure the correlations were insignificant. Only after calculation of the d’, which accounts for a possible response bias, was a significant brain-behaviour relation found. Such a response bias might be especially inherent in the processing of faces because they are stimuli of high emotional valence which might give rise to a significant number of false positive responses compared to the processing of affectively neutral object categories like houses.

### 4. Which factors distinguish the present results from previous electrophysiological studies on congenital prosopagnosia?

Previous electrophysiological studies [22–27, 52] were not able to reveal a group based difference between cPA and unimpaired subjects. Their results showed abolished as well as preserved N and M170 face selectivity [22–27]. A recent study [27] found an absence of the face inversion effect in subjects with cPA, i.e. the N170 was neither enhanced or delayed.

Surprisingly in most of the studies to date latency of the N170 or the M170 has not been taken into account. Two studies [24] and [25] concluded that the heterogeneity of results is indicative of cPA being a heterogeneous entity with different subjects being impaired at different stages of face processing. This conclusion is not possible so far. Also the present group results do not allow us to finally determine whether cPA constitutes a homogeneous entity or not. Strictly speaking this would imply revealing the deficit on an individual basis. However, one result in this study points to the fact that impairment of the M170 processing stage is a characteristic of cPA: the use of bilateral amplitude and latency values of the M170 leads to a 90% correct individual classification rate through discriminant analysis (Fisher).

In our opinion two factors may significantly contribute to the difference between the results of this study and previous ones:

*a*. *Predominant use of EEG as recording technique*. Given that the majority of existing studies [22–25, 27] [22–24, 27] used EEG as a recording technique it is remarkable that the simultaneous MEG/EEG measurement in the current study reveals that EEG in contrast to MEG is not able to detect consistent differences of peak amplitude and latency of the 170ms response to faces between unimpaired persons and prosopagnosics. The importance of the negative EEG result is its accordance with the literature under comparable measuring conditions (comparable number and spacing of electrodes [10-20-system], standard analysis of peak amplitude and latency).

It is in principle possible that a denser spacing of electrodes and the computation of a spatial average, as we have done with MEG, could increase the signal to noise ratio and reveal a difference between subjects with cPA and controls using EEG. This possibility is suggested by the fact that differences between the two groups are detected by EEG if the time course of face minus house (categorical) differences is analysed (cf. Fig 7C and 7D. Generally, it was not the main objective of this study to provide a definite explanation for the discrepancy between the EEG and MEG results. This issue, which has to take into account the differential sensitivity of MEG and EEG to sources of radial orientation, awaits future analysis that is beyond the scope of the present paper. MEG has been used in previous studies [24, 26] [25, 26] but in these studies the number of subjects was smaller compared to the present study. Five subjects with cPA were included in the study by Harris et al. [24, 25] and 6 subjects with cPA in the study by Rivolta et al. [26], which might explain the absence of a significant group effect.

*b*. *The choice of the experimental task*. A second factor that might have influenced results of existing studies is the choice of the task to sustain attentive fixation. For this purpose in all EEG studies (see above), except for Towler et al. 2012 [27], object discrimination or detection tasks have been used. Such manipulations could drive object-related neural processing. Notably, in Bentin, Deouell & Soroker 1999, [22] and Kress and Daum 2003, [23] the reduced amplitude difference between the N170 evoked by faces and houses is attributed to elevated object (house) related activity. This impact of task might also explain why a recent study found that the N170 response of three members of the same family with cPA is almost similar to faces and their Fourier-phase randomized counterparts (noise) [53].

In our study the loss of face selectivity is due to reduced amplitude of the face-evoked M170 whereas object-related activity stays unchanged (Fig 6B). The reduction is on average 45% which is large but not complete. This is important because a recent study [26] found no significant difference in face selectivity (calculated as the difference between average MEG activity for faces and places) between a groups of six subjects with cPA and 11 controls. The relevance of group size is highlighted in Fig 2 in [26]: the average MEG activity to faces in the right fusiform area is approximately 9 (a complex measure to approximate source current strength used by the authors, cf. methods) compared to 6 in subjects with cPA. Unfortunately the authors do not report whether this difference is significant. Apart from that, the magnitude of the difference is well in the order of what is reported here (roughly 30%). Also, it can be clearly seen in Fig 2 in [26] that the peak of the M170 is delayed in subjects with cPA.

The following conclusions can be drawn, which confirm our working hypotheses:

1. Subjects with cPA show a selective deficit in familiar face processing whereas extensive behavioural testing reveals no impairment in any other domain.
2. This behavioural result is in agreement with a category (face) selective alteration of the M170 processing stage.
3. This category-selective alteration can be found in a passive viewing task and in a second task which requires the subject to hold a single face or house in working memory and selectively respond to it. This means that the category-specific alteration of the M170 seems to occur independently of top-down demands. This is compatible with impaired early mandatory (holistic) face processing.
4. A differential brain-behaviour correlation of the size of the M170 amplitude and face recognition performance is indicative of functional significance of altered M170 in subjects with cPA.

In summary, our results present evidence that corroborates the view of modularization of early perceptually-based face processing stages. In such a model congenital prosopagnosia and unimpaired face processing represent two sides of the same coin.

## Supporting Information

S1 Fig Results of the FFHRT: analysis of dprime (d’) and correlational analysis. A. Like for accuracy (Fig 4 in the main paper) d’-measures for famous face recognition are nearly significantly different between groups ((***p = 0*.*054 (3*.*694)***); black circles: controls; red squares: people with cPA) whereas object recognition is indistinguishable (***p = 0*.*424(0*.*638)***; green circles: controls; blue squares: people with cPA). B. The left panel shows scatter plots which relate accuracy (percent correct) for famous faces and houses (black-green circles: controls; red-blue squares: people with cPA). The right panel analogously displays scatter plots for reaction time. Accuracy measures for face and object recognition are uncorrelated in both groups (controls: ***r* = 0.276, *p* = 0.300; cPA: *r* = 0.303, *p* = 0.315**). This indicates at least partial independence of face and object processing and is not compatible with a general object recognition failure in cPA. Reaction time measures are highly correlated between categories which suggests that reaction time is a marker of individual response tendency independent of task performance (**controls: *r* = 0.904, *p <*0.001; cPA: *r* = 0.632, *p* = 0.021**).
(TIF)

S2 Fig Scatter plots of M170 peak amplitudes in paradigm 1 and 2. The scatter plots show the excellent correspondence between the main experiment and the control, here visualized for the condition without attention. Upper panels: amplitudes of face evoked M170 (black circles: controls; red squares: people with cPA). Lower panels: amplitude of house evoked M170 (green circles: controls; blue squares: people with cPA).
(TIF)

S3 Fig Scatter plots of M170 peak latencies in paradigm 1 and 2. Colour coding identical to S2 Fig, except the fact that now peak latencies have been plotted.
(TIF)

S1 Table Neuropsychological tests for controls and individuals with Prosopagnosia. The majority of tests give results which are indistinguishable between groups. For explanation of each individual test measure see above (description of neuropsychological assessment). Verbal IQ and overall IQ are different between groups which is paralleled by the fact that their educational level is different (cf. Online Methods). However, the group means for persons with cPA are well above the general mean. Also, the block span in the blocktapping task is relatively longer for the control group. In absolute values, persons with cPA score in the normal range as a block span of 5 is considered normal. In addition, performance in various tests of object recognition is indistinguishable between both groups.
(DOC)

S2 Table Results of the Famous Face/House Recognition Test (FFHRT). Reaction time (ms) and accuracy (percent correct) are significantly different for famous faces between groups whereas performance for unfamiliar faces and houses is statistically indistinguishable.
(DOC)

S3 Table Bivariate Pearson’s Correlations between different tests of face perception and recognition. Performance measures in the BFRT, the CMTF and the FFHRT are significantly correlated whereas performance in the RMT only shows a significant correlation with the BFRT. Numbers without brackets denote correlation coefficients; those with brackets denote level of significance.
(DOC)

S4 Table Bivariate Pearson’s Correlations between M170 amplitudes and latencies in paradigm 1 and 2. As can be taken from S2 and S3 Figs controls and people with cPA scatter around the diagonal. Therefore, as an indicator for the general stability of results in the control experiment the correlations were computed for both groups together. Most resulting correlations are of high strength and significance. Correlations between house evoked M170 latencies are slightly weaker, the reason being that due to smaller amplitudes of house evoked M170 amplitudes in some instances the peaks were less well defined which resulted in higher estimation uncertainty and error.
(DOC)

S1 Text Description of neuropsychological assessment. (DOC)

## Abbreviations

apA: acquired prosopagnosia
cPA: congenital prosopagnosia
BESA: Brain Electrical Source Analysis
BFRT: Benton Facial Recognition Test
CMTF: Cambridge Memory Test for Faces
d’: d-prime
DTI: diffusion tensor imaging
EEG: Electroencephalography
EOG: Electrooculography
ERF: event related field
ERP: event related potential
FFHRT: Famous Face/House Recognition Test
fMRI: functional magnetic resonance imaging
ICA: independent component analysis
IQR: Interquartile range
ISI: interstimulus interval
MEG: Magnetoencephalography
RMT: Warrington Recognition Memory Test
