# Supplementary figures and images for: The 170ms Response to Faces as Measured by MEG (M170) Is Consistently Altered in Congenital Prosopagnosia
Source: PLoS One. 2015 Sep 22;10(9):e0137624. doi: 10.1371/journal.pone.0137624 (PMC4579010; doi:10.1371/journal.pone.0137624)

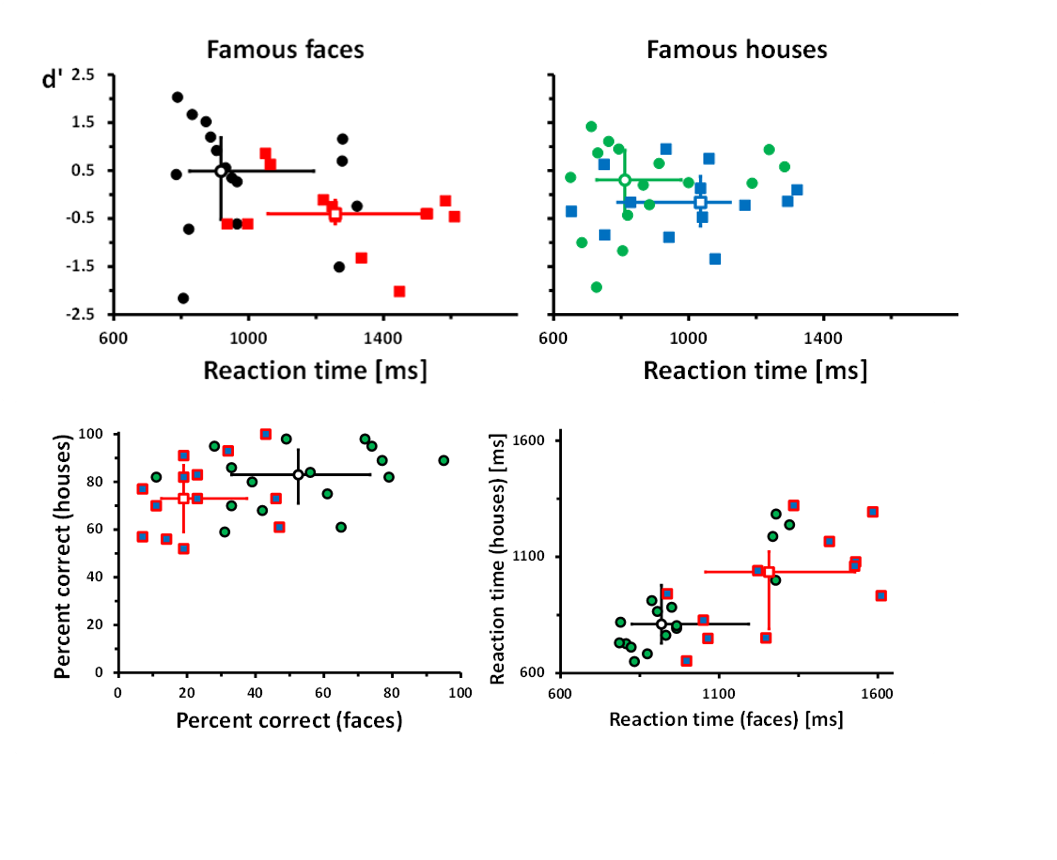

Supplement: S1 Fig — A. Like for accuracy (Fig 4 in the main paper) d’-measures for famous face recognition are nearly significantly different between groups ((p = 0.054 (3.694)); black circles: controls; red squares: people with cPA) whereas object recognition is indistinguishable (p = 0.424(0.638); green circles: controls; blue squares: people with cPA). B. The left panel shows scatter plots which relate accuracy (percent correct) for famous faces and houses (black-green circles: controls; red-blue squares: people with cPA). The right panel analogously displays scatter plots for reaction time. Accuracy measures for face and object recognition are uncorrelated in both groups (controls: r = 0.276, p = 0.300; cPA: r = 0.303, p = 0.315). This indicates at least partial independence of face and object processing and is not compatible with a general object recognition failure in cPA. Reaction time measures are highly correlated between categories which suggests that reaction time is a marker of individual response tendency independent of task performance (controls: r = 0.904, p <0.001; cPA: r = 0.632, p = 0.021). (TIF) [file pone.0137624.s001.tif]

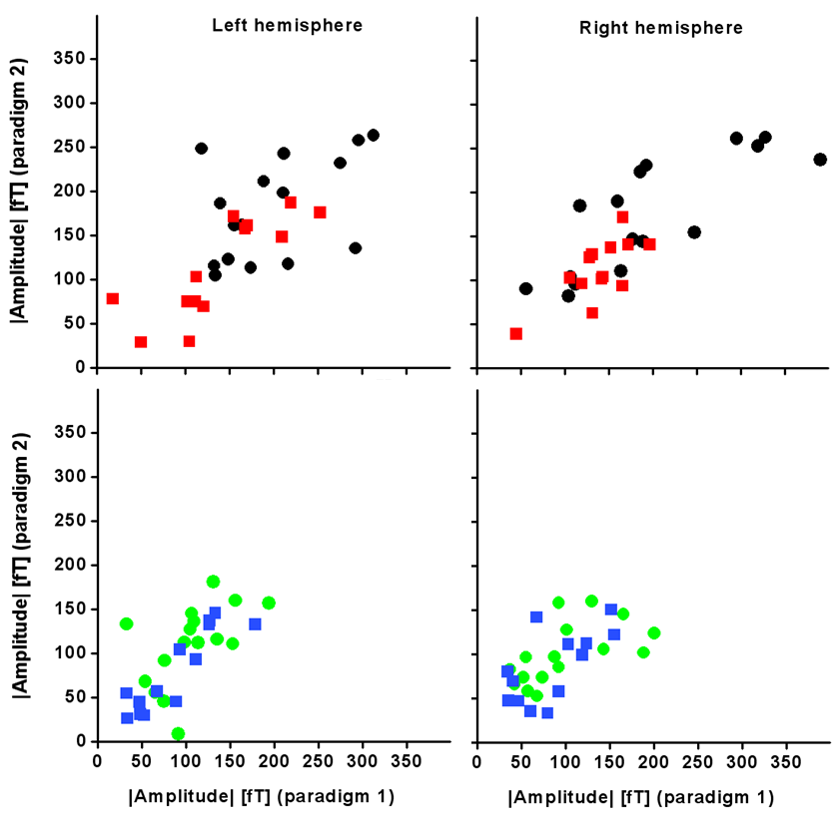

Supplement: S2 Fig — The scatter plots show the excellent correspondence between the main experiment and the control, here visualized for the condition without attention. Upper panels: amplitudes of face evoked M170 (black circles: controls; red squares: people with cPA). Lower panels: amplitude of house evoked M170 (green circles: controls; blue squares: people with cPA). (TIF) [file pone.0137624.s002.tif]

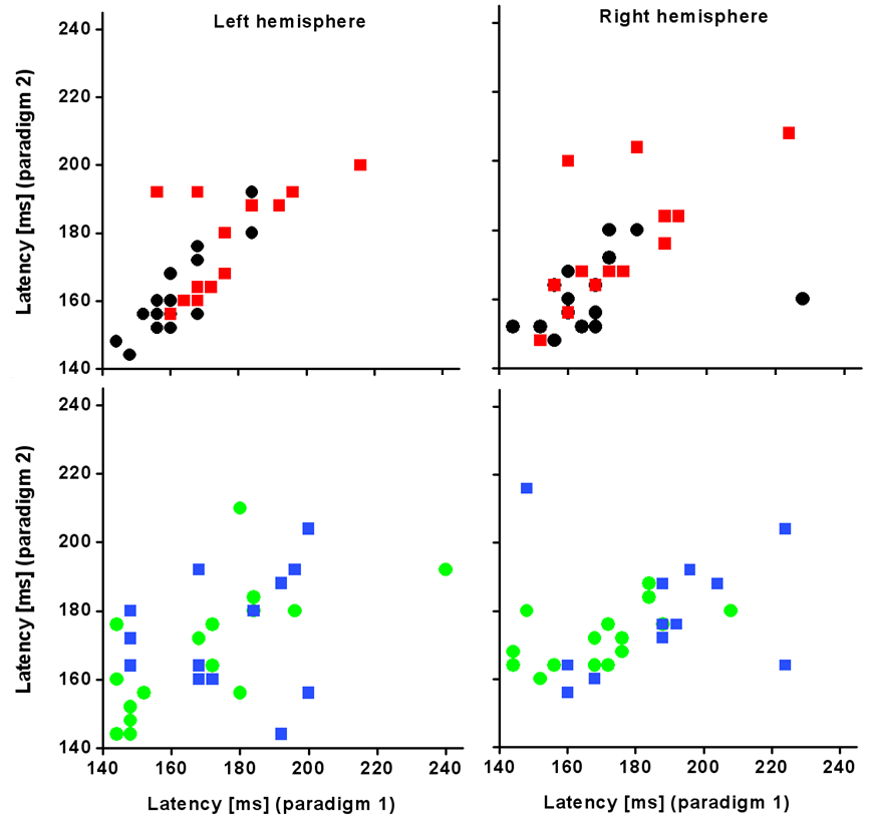

Supplement: S3 Fig — Colour coding identical to S2 Fig, except the fact that now peak latencies have been plotted. (TIF) [file pone.0137624.s003.tif]
