# Supplementary material for: The 170ms Response to Faces as Measured by MEG (M170) Is Consistently Altered in Congenital Prosopagnosia
Source: PLoS One. 2015 Sep 22;10(9):e0137624. doi: 10.1371/journal.pone.0137624 (PMC4579010; doi:10.1371/journal.pone.0137624)
Supplement: S1 Table — The majority of tests give results which are indistinguishable between groups. For explanation of each individual test measure see above (description of neuropsychological assessment). Verbal IQ and overall IQ are different between groups which is paralleled by the fact that their educational level is different (cf. Online Methods). However, the group means for persons with cPA are well above the general mean. Also, the block span in the blocktapping task is relatively longer for the control group. In absolute values, persons with cPA score in the normal range as a block span of 5 is considered normal. In addition, performance in various tests of object recognition is indistinguishable between both groups. (DOC) [file pone.0137624.s004.doc]

| **Test** | **Controls**  **(mean [IQR])** | **cPA**  **(mean [IQR])** | **sign. (Wald-Chi-square)** |
| --- | --- | --- | --- |
| **Freiburg Visual Acuity Test** | 0.619[0.075] | 0.566[0.080] | **0.587 (0.295)** |
| **Contrast sensitivity**  Pelli-Robson left eye  Pelli-Robson right eye  Pelli-Robson binocular | 1.89[0.15]  1.85[0.30]  1.95[0.00] | 1.85[0.15]  1.81[0.30]  1.94[0.00] | **0.387 (0.747)**  **0.256 (1.288)**  **0.649 (0.207)** |
| **Intelligence:** Verbal IQ Performance IQ Overall IQ | 133.4[15.0] 123.1[13.5] 135.1[12.5] | 122.1[13.5]  123.2[16.5]  126.8[12.5] | **0.020 (5.399) 0.954 (0.003)**  **0. 123 (2.377)** |
| **Visual memory:** Benton visual retention test (**10**)  Rey complex figure age scaled  Rey complex figure (30 min delay) Blocktapping | 8.4[1.0]  71.9[0.0]  49.9[14.3]  6.44[1.00] | 7.9[1.5]  70.2[3.0]  41.1[13.5]  5.50[1.00] | **0.724 (0.124)**  **0.009 (6.694)**  **0.073 (3.216)**  **0.001 (10.985)** |
| **Spatial perception:** Benton Judgement of Line Orientation (**30**) | 27.8[3.0] | 26.5[5.5] | **0.305 (1.052)** |
| **object recognition:** BNT (**60**) Hooper VOT (**30**)  VOSP (letters, **20**)  VOSP (silhouettes, **30**)  VOSP (object recognition, **20**) | 57.56[2.75]  26.3[2.0]  17.0[2.75]  22.4[2.0]  14.6[4.3] | 57.69[2.000]  26.9[1.25]  16.5[7.0]  21.6[3.0]  12.9[3.0] | **0.874 (0.025) 0.343 (0.898)**  **0.556 (0.346)**  **0.414 (0.667)**  **0.093 (2.829)** |
