# Supplementary material for: The 170ms Response to Faces as Measured by MEG (M170) Is Consistently Altered in Congenital Prosopagnosia
Source: PLoS One. 2015 Sep 22;10(9):e0137624. doi: 10.1371/journal.pone.0137624 (PMC4579010; doi:10.1371/journal.pone.0137624)
Supplement: S2 Table — Reaction time (ms) and accuracy (percent correct) are significantly different for famous faces between groups whereas performance for unfamiliar faces and houses is statistically indistinguishable. (DOC) [file pone.0137624.s005.doc]

| **Parameter** | **Controls** | **cPA** | **sign. (Wald-Chi-square)** |
| --- | --- | --- | --- |
| Unfamiliar Houses (RT)  Unfamiliar Houses (Percent Correct) | 1028.12[297.43] 80.0[16] | 1171.02[321.88]  84.4[22] | **0.276(1.187) 0.152(2.054)** |
| Famous Houses (RT) Famous Houses (Percent Correct) | 878.46[249.85] 81.9[22] | 988.66[332.88]  74.0[28] | **0.138(2.199) 0.175(1.841)** |
| Unfamiliar Faces (RT) Unfamiliar Faces (Percent Correct) | 920.08[277.98] 94.1[9] | 1025.97[339.00]  96.5[5] | **0.154(2.033)**  **0.221(1.497)** |
| Famous Faces RT Famous Faces (Percent Correct) | 978.95[368.09] 52.8[41] | 1293.03[471.43] 23.8[25] | **<0.001(18.558) <0.001(17.950)** |
