# Supplementary material for: The 170ms Response to Faces as Measured by MEG (M170) Is Consistently Altered in Congenital Prosopagnosia
Source: PLoS One. 2015 Sep 22;10(9):e0137624. doi: 10.1371/journal.pone.0137624 (PMC4579010; doi:10.1371/journal.pone.0137624)
Supplement: S3 Table — Performance measures in the BFRT, the CMTF and the FFHRT are significantly correlated whereas performance in the RMT only shows a significant correlation with the BFRT. Numbers without brackets denote correlation coefficients; those with brackets denote level of significance. (DOC) [file pone.0137624.s006.doc]

|  | **Correct hits on Famous faces (FFHRT)** | **BFRT** | **RMT** | **CMTF Upright** | **CMTF**  **Inverted** |
| --- | --- | --- | --- | --- | --- |
| **Reaction time on Famous faces (FFHRT) (median)** | **-0.431(0.020)** | **-0.519(0.003)** | **-0.545(0.002)** | **-0.704(<0.001)** | **-0.458(.016)** |
| **Correct hits on Famous faces (FFHRT)** |  | **0.401(0.031)** | **0.282 (.137)** | **0.662 (<.001)** | **0.564 (.002)** |
| **BFRT** |  |  | **0.474 (.009)** | **0.504 (.006)** | **0.668 (<.001)** |
| **RMT** |  |  |  | **0.280 (.150)** | **0.394 (.042)** |
| **CMTF Upright** |  |  |  |  | **0.541 (.003)** |
