# Supplementary material for: The 170ms Response to Faces as Measured by MEG (M170) Is Consistently Altered in Congenital Prosopagnosia
Source: PLoS One. 2015 Sep 22;10(9):e0137624. doi: 10.1371/journal.pone.0137624 (PMC4579010; doi:10.1371/journal.pone.0137624)
Supplement: S4 Table — As can be taken from S2 and S3 Figs controls and people with cPA scatter around the diagonal. Therefore, as an indicator for the general stability of results in the control experiment the correlations were computed for both groups together. Most resulting correlations are of high strength and significance. Correlations between house evoked M170 latencies are slightly weaker, the reason being that due to smaller amplitudes of house evoked M170 amplitudes in some instances the peaks were less well defined which resulted in higher estimation uncertainty and error. (DOC) [file pone.0137624.s007.doc]

|  | **Latency of the M170** | | **Amplitude of the M170** | |
| --- | --- | --- | --- | --- |
|  | **left** | **right** | **left** | **right** |
| **Faces** | **.820 (<0.001)** | **.567(0.001)** | **.726 (<0.001)** | **.802 (<0.001)** |
| **Houses** | **.122 (0.530)** | **.212 (0.270)** | **.649 (p<0.001)** | **.663 (<0.001)** |
