# Supplementary material for: The 170ms Response to Faces as Measured by MEG (M170) Is Consistently Altered in Congenital Prosopagnosia
Source: PLoS One. 2015 Sep 22;10(9):e0137624. doi: 10.1371/journal.pone.0137624 (PMC4579010; doi:10.1371/journal.pone.0137624)
Supplement: S1 Text — (DOC) [file pone.0137624.s008.doc]

**S1 Text. Description of neuropsychological assessment**

A large battery of tests was administered to both control and prosopagnosic participants. The tests will be grouped according to the visual and cognitive functions they assess.

Colour vision was tested with the **L´Anthony D15 test**. This test is a modification of the Farnsworth-Munsell 100 Hue test . It allows a quick classification, and even mild deficits in colour vision can be detected. The participant is prompted to sequentially arrange 15 unsaturated coloured discs. Both groups scored in the normal range (controls: 10 subjects without error; 6 subjects with 1 error (commutation of adjacent discs); persons with cPA: 8 subjects without error; 5 subjects with 1 error (commutation of adjacent discs)).

Visual acuity was measured with the **Freiburg Visual Acuity Test** which is an automa-ted measurement of visual acuity by using Landolt-Cs with different gap size. Here, visual acuity is expressed as the minimum angle of resolution in minutes of arc.

Contrast sensitivity was assessed with the **Pelli-Robson Contrast Sensitivity Chart** . In this test, the participant has to read with both eyes rows of letters that are grouped into triplets where the contrast diminishes from one triplet to the next. The triplet where the participant is able to read at least two of the three letters is taken to assess the Log contrast sensitivity.

Intelligence was tested with the **Hamburg-Wechsler Intelligenztest** - Revised (HAWIE-R,). The test is a German adaption of the Wechsler Adult Intelligence Scale Revised (WAIS-R). The HAWIE-R allows to separately asses a verbal and a performance-IQ, and the calculation of an overall IQ-score.

Object recognition was examined with three tests:

1. The **Visual Object and Space Perception Battery** (VOSP;) consists of eight tests each designed to assess a particular aspect of object or space perception.

We used three subtests:

a) Incomplete letters

Participants have to identify 20 letters which are covered at about 70% by a random dot pattern.

b) Silhouette

Participants have to identify non-canonical views of 15 animals and 15 common objects (black silhouettes).

c) Object recognition

Participants have to identify the one real object out of four presented on 20 sheets total. Also here, the objects were represented by three-dimensional black silhouettes.

2. The **Boston Naming Test** (BNT;), a standard test of object naming. Sixty line drawings of objects ranging from high-frequency words such as a house to rare ones such as an abacus have to be named. The test score results from the number of correctly named items.

3. The **Hooper Visual Object Organization Test** (VOT; ).

 This test consists of 30 line drawings, each showing a common object - such as an apple or a ball - that has been cut into several pieces. The pieces are scattered on the page like parts of a puzzle. The task is to tell what the object would be if the pieces were rearranged correctly.

Visual memory was tested with two tests:

1. The **Benton Visual Retention Test** measures immediate free recall.

10 drawings of simple geometrical figures are presented each for 10 seconds (encoding phase). After removal of the drawing, the subject is prompted to draw it immediately from memory. This test does not only measure memory for geometrical figures, but also visuo-constructive abilities. The number of correctly reproduced items was scored.

2. The **Corsi Block Tapping Task**

It measures visuo-spatial working memory capacity (span).

The subject has to mimic the investigator as he randomly taps nine identical spatially separated blocks. The sequence starts out using three blocks and increases by one until the subject is no longer able to repeat two sequences without error. This number is known as the Corsi (block) span and averages 5 for normal human individuals.

Spatial perception was assessed with the **Benton Line Orientation Test** . Two lines of different orientations on the top of the page (the test) have to be compared to a fan-shaped set of 11 lines of different orientations on the bottom of the page and the subject has to name the corresponding lines of identical orientation. As there are 30 test pairs the maximum score is 30.

Face perception and recognition were assessed with four different tests:

First, two common standardized tests of face recognition, the **Benton Facial Recognition Test** (BFRT; ) and the **Warrington Recognition Memory Test** (RMT; ) were used. In the BFRT, the participant matches a stimulus picture to six response-choice pictures which are shown simultaneously. The Long Form of the test was applied. The score results from the number of correct matches.

The RMT consists of a set of 50 face photographs. In the encoding phase, the subject has to evaluate each picture in terms of sympathy without being told that the faces have to be recognized later (implicit learning). In the test phase, each of the faces from the encoding phase (target) is presented together with a new face (non-target) and the subject has to point to the target.

As a computerized test of face learning and recognition, the recently developed **Cambridge Memory Test for Faces** (CMTF; ) was used. It has three subtests. In the first one, a face is presented and has to be subsequently recognized among three faces. Because of preceding instruction trials, the CMTF probes explicit learning. In this manner 6 faces are learned. Each of these learned faces has to be recognized in the second subtest amongst 6 faces. The third subtest is identical to the second, apart from the fact that now the visibility of all stimuli is degraded by overlaid noise. Both versions of the test, i.e. upright and inverted face presentation, were applied. The CMTF has proven to allow a good separation between prosopagnosic and non-prosopagnosic individuals .

Finally, in a self-made computerized test (**Famous Face/House Recognition Test**, FFHRT) the recognition of famous faces and houses was assessed: each stimulus was presented until button press and for maximally 4 seconds in a random sequence of famous or unknown faces and houses (50 famous and 50 unknown stimuli in each category). In the inter-stimulus-interval (ISI, length: 500 ms) a grey background was shown with a fixation cross. The participant was prompted to judge both as quickly and as accurately as possible whether the particular stimulus was famous or unknown. Accuracy (percent correct) and reaction times for the correctly judged items were analysed.

**References:**

1. Farnsworth D (1943) The Farnsworth-Munsell 100-Hue and Dichotomous Tests for Color Vision. J Opt Soc Am 33: 568-574.

2. Bach M (1996) The Freiburg Visual Acuity test--automatic measurement of visual acuity. Optom Vis Sci 73: 49-53.

3. Pelli D, Robson J, Wilkins A (1988) The design of a new letter chart for measuring contrast sensitivity. Clin Vision Sci 2: 187–199

4. Tewes U (1991) HAWIE-R: Hamburg-Wechlser Intelligenztest für Erwachsene Revision 1991. Handbuch und Testanweisung. Bern: Verlag Hans Huber.

5. Lezak MD, Howieson DB, Loring DW, Hannay HJ, Fischer JS (2004) Neuropsychological Assessment. Oxford: Oxford University Press.

6. Kaplan E, Goodglass H, Weintraub S (1983) The Boston Naming Test. Philadelphia: Lea & Felbinger.

7. Hooper HE (1983) The Hooper Visual Organization Test Manual. Los Angeles: Western Psychological Services.

8. Benton Sivan A, Spreen O (1996) Der Bentontest. . Bern: Verlag Hans Huber.

9. Corsi PM (1972) Human memory and the medial temporal region of the brain. Montreal: McGill University.

10. Benton AL, Hamsher KdS, Varney NR, Spreen O (1983) Contributions to Neuropsychological Assessment: A Clinical Manual. New York: Oxford University Press.

11. Warrington E (1984) Recognition Memory Test. Manual. Los Angeles: Western Psychological Services.

12. Duchaine B, Nakayama K (2006) The Cambridge Face Memory Test: results for neurologically intact individuals and an investigation of its validity using inverted face stimuli and prosopagnosic participants. Neuropsychologia 44: 576-585.
